# Extracellular Vesicles: Emerging Players in Plant Defense Against Pathogens

**DOI:** 10.3389/fpls.2021.757925

**Published:** 2021-09-30

**Authors:** Guosheng Liu, Guangren Kang, Shumei Wang, Yifan Huang, Qiang Cai

**Affiliations:** ^1^State Key Laboratory of Hybrid Rice, College of Life Science, Wuhan University, Wuhan, China; ^2^Hubei Hongshan Laboratory, Wuhan, China; ^3^Department of Microbiology and Plant Pathology, Center for Plant Cell Biology, Institute for Integrative Genome Biology, University of California, Riverside, Riverside, CA, United States

**Keywords:** extracellular vesicles, cell-to-cell communication, plant immunity, cross-kingdom RNA interference, endomembrane trafficking

## Abstract

Communication between plants and interacting microorganisms requires functional molecule trafficking, which is essential for host defense and pathogen virulence. Extracellular vesicles (EVs) are single membrane-bound spheres that carry complex cargos, including lipids, proteins, and nucleic acids. They mediate cell-to-cell communication *via* the transfer of molecules between cells. Plant EVs have been isolated from many plant species and play a prominent role in immune system modulation and plant defense response. Recent studies have shown that plant EVs are emerging players in cross-kingdom regulation and contribute to plant immunity by mediating the trafficking of regulatory small RNA into pathogens, leading to the silencing of pathogen virulence-related genes. This review summarizes the current understanding of plant EV isolation technologies, the role of plant EVs in plant immunity, and the mechanism of plant EV biogenesis, as well as approaches for how these findings can be developed into innovative strategies for crop protection.

## Introduction

Numerous plant pathogens, including bacteria, fungi, and nematodes, are responsible for many plant diseases, which reduce the yield and quality of agricultural production worldwide every year (Fisher et al., 2012; Savary et al., 2019). Exploring the interaction between plants and pathogens is conducive to plant disease control and agricultural production. Plants and pathogens secrete multitudes of molecules into the extracellular environment for cross-border communication, which is crucial to plant defense and pathogen virulence (Delaunois et al., 2014; Toruno et al., 2016). Based on our current understanding, extracellular vesicles (EVs) represent a major way to achieve this communication (Cai et al., 2021).

EVs are single membrane-bound heterogeneous spheres that are released by cells into the extracellular space (Colombo et al., 2014). They contain a diverse variety of enclosed bioactive cargos, such as proteins, nucleic acids, and metabolites (Colombo et al., 2014). EVs are currently categorized as exosomes, microvesicles, and apoptosis-derived vesicles on the basis of their origins and sizes (van Niel et al., 2018). Exosomes, which have diameters ranging from 30nm to 150nm, are derived from multivesicular bodies (MVBs) after fusing with the plasma membrane to release their intraluminal vesicles (ILVs; Colombo et al., 2014). Microvesicles normally refer to 150–1,000nm vesicles, which are shed from the plasma membrane during cell stress (Heijnen et al., 1999; Colombo et al., 2014; van Niel et al., 2018). Apoptosis-derived vesicles, which are characterized by their large size range of 30–10,000nm, result from cell apoptosis (Atkin-Smith et al., 2015).

In the 1980s, EVs were initially thought to be a disposal mechanism for waste removal from cells (Johnstone et al., 1987). However, decades of studies have shown that numerous active molecules are transported by EVs and are featured in various biological processes, including cellular communication, immune response, antigen presentation, and cancer cell migration (Colombo et al., 2014). The latest studies have indicated that plant-released EVs play a major role in transboundary communication between plants and pathogens (Cai et al., 2018b, 2019, 2021). However, owing to the limitations of EV isolation methods, the research on plant EVs is only beginning.

## Plant Evs

**Figure 1:**
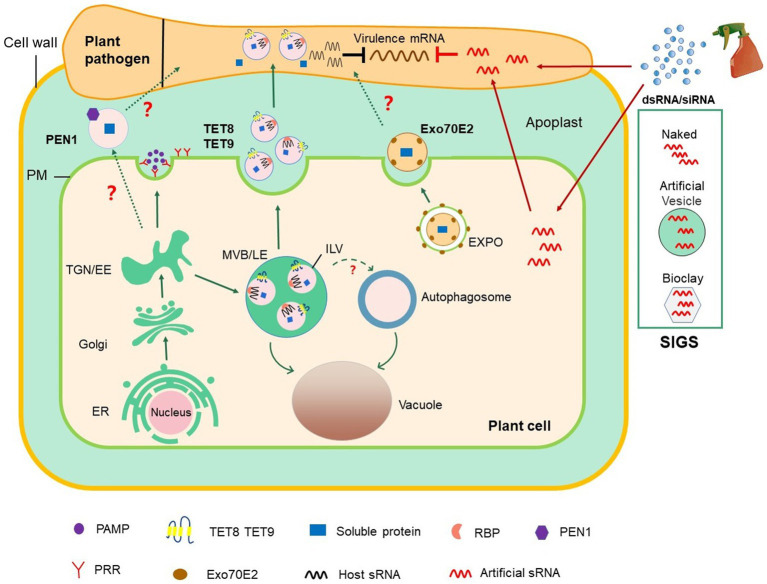
Roles of EV-mediated RNAi in plant–microbial interactions and plant protection. The conventional secretion pathway delivers PAMPs to the extracellular space or transport surface PRRs to PM. In unconventional secretion pathway, MVBs release TET8/9-positive EVs, which contain defense proteins and host-derived sRNAs, into the extracellular space. TET8/9-positive EVs contain a variety of RBPs, including AGO1, RHs, and ANNs, which load sRNAs into EVs. PEN1-positive EVs are secreted into the extracellular space with an unknown mechanism (marked with? in the Figure). EXPO is a novel bilayer membrane organelle that fuses with PM to produce another subtype of EVs. Whether PEN1-positive and EXPO-positive EVs contribute to cross-kingdom RNAi between plant and pathogens (marked with? in the figure) is unknown. On the basis of the knowledge on cross-kingdom RNAi, the spray application of dsRNAs and sRNAs that target pathogen genes can potentially control plant diseases. In SIGS approaches, dsRNAs are applied exogenously or carried by nanocarriers, such as BioClay and artificial vesicles. Exogenous RNAs can either be directly internalized into fungal cells or indirectly *via* passage through plant cells before transport into fungal cells. dsRNA, double-stranded RNA; EE, early endosome; ER, endoplasmic reticulum; EVs, extracellular vesicles; EXPO, exocyst-positive organelle; MVB/LE, multivesicular body/late endosome; ILV, intraluminal vesicle; PAMP, pathogen-associated molecular pattern; PEN1, Penetration 1; PM, plasma membrane; PRR, pattern recognition receptor; RBPs, RNA binding protein; SIGS, spray-induced gene silencing; siRNA, small interfering RNA; sRNA, small RNA; TGN, *trans*-Golgi network.

EVs widely exist in eukaryotes, and numerous studies have shown that in animals, EVs are heterogeneous groups that encompass diverse subclasses and perform different functions (Kowal et al., 2016; Jeppesen et al., 2019). In plants, EVs have been isolated and purified from apoplastic washing fluid (AWF) collected from leaves and seeds or from pollen germination media (Cai et al., 2021; Table 1). At least three different subtypes of EVs have been characterized in *Arabidopsis* by taking advantage of their markers: tetraspanin (TET) 8, penetration 1 (PEN1), and exocyst subunit Exo70 family protein (Exo70) E2 (Wang et al., 2010; Rutter and Innes, 2017; Cai et al., 2018b; Figure 1).

**Table 1:** List of the protein markers, confirmed cargoes, isolation methods, and biological functions of various EVs isolated from plants.

Plant	EV marker	EV Cargo	Function	Isolation method	Method advantages	Method disadvantages	Ref.
*Arabidopsis*	TET8	sRNAs, RBPs	Transport sRNAs to *B. cinerea* to silence virulence genes	Differential centrifugation	Enable isolation from large volumes	Unable to separate different EVs	Cai et al., 2018b, He et al., 2021
				Density gradient centrifugation	Enable separate EV sub-types	Unable to separate EVs with similar density	
				Immunoaffinity capture	High purity, high selectivity	Require specific antibodies	
*Arabidopsis*	PEN1	PATL1, PATL2	Enriched in plant defense components	Differential centrifugation	Enable isolation from large volumes	Unable to separate different EVs	Rutter and Innes, 2017
				Density gradient centrifugation	Enable separate EV sub-types	Unable to separate EVs with similar density	
Sunflower	nd	PMR5, GDSL, Lectins	Antifungal activity, enriched in cell wall enzymes	Differential centrifugation	Enable isolation from large volumes	Unable to separate different EVs	Regente et al., 2017, de la Canal and Pinedo, 2018
Olive	Ole e1, Ole e11, Ole e12	PCBER, GADPH	Secreted during pollen germination and pollen tube growth	Differential centrifugation	Enable isolation from large volumes	Unable to separate different EVs	Prado et al., 2014
				Density gradient centrifugation	Enable separate EV sub-types	Unable to separate EVs with similar density	
*N. benthamiana*	nd	HSP70, AGO2	Release virus components	Differential centrifugation	Enable isolation from large volumes	Unable to separate different EVs	Movahed et al., 2019

*AGO2, Argonaute 2; GADPH, glyceraldehyde 3-phosphate dehydrogenase; HSP70, heat shock 70kDa protein; n.d., no date (reference materials); PATL1, PATELLIN 1; PATL2, PATELLIN 2; PEN1, Penetration 1; RBPs, RNA binding protein; PCBER, phenylcoumaran benzylic ether reductase PT1; PMR5, powdery mildew resistance protein 5; sRNA, small RNA; TET8, tetraspanin 8*.

Animal TETs, such as CD9, CD81, and CD63, are highly enriched in the membranes of exosomes and therefore serve as exosome markers (Escola et al., 1998; Simpson et al., 2012; Andreu and Yanez-Mo, 2014; Kowal et al., 2016). TET8 and TET9 are plant homologs of animal TET proteins (Cai et al., 2018b). TET8 and TET9-positive vesicles partially colocalize with the MVB marker Rab5-like guanosine triphosphatase ARA6 (also known as RABF1) inside of cells, and TET8-positive EVs have been observed outside of cells (Cai et al., 2018b; He et al., 2021). Thus, TET8-positive EVs are considered as plant exosomes. Furthermore, evidence has shown that the expression of *TET8* can be induced by *Botrytis cinerea* infection, and TET8-positive vesicles have been observed to accumulate at *B. cinerea* infection sites (Cai et al., 2018b), showing that TET8-associated EVs are involved in response to pathogen attack.

PEN1 has been previously shown to mediate trafficking between the Golgi complex and the plasma membrane (Kwon et al., 2008). PEN1 is also present in EVs induced by bacterial pathogen infection or salicylic acid treatment (Rutter and Innes, 2017). The secretion of PEN1 is dependent on ADP ribosylation factor-GTP exchange factor GNOM (Nielsen et al., 2012), and PEN1 does not colocate with ARA6, indicating that the biogenic pathway of TET8-positive EVs is different from that of PEN1-positive EVs (He et al., 2021). Moreover, EVs isolated from transgenic plants co-express two fluorescence-tagged fusion proteins, TET8-GFP and mCherry-PEN1, and display two distinct GFP-labeled and mCherry-labeled EVs (He et al., 2021). These characteristics confirm that PEN1-positive EVs and TET8-positive EVs are two subtypes of EVs (He et al., 2021).

Exocyst-positive organelle (EXPO) is a novel organelle that is identified by using live cell imaging and immunogold labeling in plants (Wang et al., 2010; Ding et al., 2014). Although EXPO and autophagosomes are bilayer structures, they do not co-locate with each other except in vacuoles upon autophagic induction (Wang et al., 2010; Lin et al., 2015). EXPO has been observed to fuse with the plasma membrane and deliver Exo70E2-positive EVs into the extracellular space. The secretory pathway of Exo70E2 is independent of MVB pathways, and EXPO is unaffected by secretory and endocytosis inhibitors in protoplasts (Wang et al., 2010; Ding et al., 2014).

## Techniques For Plant Ev Isolation

The isolation of plant EVs remains a challenge (Liu et al., 2020b). In contrast to animal EVs, which are isolated from biofluids, plant EVs are isolated from AWF (Colombo et al., 2014). Currently, a simple well-established infiltration–centrifugation method is widely used for plant AWF collection (Wang et al., 2005; Sanmartin et al., 2007; Hatsugai et al., 2009; O’Leary et al., 2014). The detached leaf protocol is the ideal method for collecting AWF before EV isolation (O’Leary et al., 2014; Madsen et al., 2016; Cai et al., 2018b; He et al., 2021). This protocol has the merit of the removal of the distinct proximal (petiole) parts of leaves. This approach could remove irrelevant RNAs in the phloem stream (Zhang et al., 2009; Liu and Chen, 2018). In addition, the leaves are supported and unlikely to be squeezed with each other during centrifugation (He et al., 2021).

Differential centrifugation is commonly used for EV isolation from plants (Rutter and Innes, 2017; Cai et al., 2018b; Liu et al., 2020b; He et al., 2021). In this method, dead cells, cell debris, and large vesicles are removed through low-velocity centrifugation at 2,000× *g* and 10,000× *g*, and the separation rate is then progressively increased to 100,000× *g* to pellet small plant EVs (Prado et al., 2014; Cai et al., 2018b; Liu et al., 2020b; He et al., 2021). However, the low centrifugal force of 40,000× *g* has also been used for the final pelleting of EVs derived from *Arabidopsis* and sunflower seeds and seedlings (Regente et al., 2009, 2017; Rutter and Innes, 2017). Notably, the separation efficiency for the isolation of TET8-positive EVs obtained by centrifugation at 100,000× *g* is much higher than that obtained by centrifugation at 40,000× *g* (He et al., 2021).

Given that plant EVs are heterogeneous populations of nanosized membrane vesicles, researchers have developed additional separation methods based on EV density and specific EV markers. Currently, the most common and practical methods for separating the subtypes of plant EVs are density gradient centrifugation and immunoaffinity capture-based techniques (Rutter and Innes, 2017; He et al., 2021). In density gradient centrifugation, sucrose and iodixanol are used as the classical media (Kowal et al., 2016; Paolini et al., 2016; Rutter and Innes, 2017; He et al., 2021). Through separation by density gradient centrifugation, TET8-positive exosomes are enriched in the gradient fraction of approximately 1.12–1.19gml^−1^, whereas PEN1-positive EVs are enriched in the gradient fraction of 1.029–1.056gml^−1^ (Rutter and Innes, 2017; He et al., 2021).

Although density gradient centrifugation can yield high-quality EVs, different EV types may have similar physical properties. Separating EVs by immunoaffinity with EV markers may be needed. Immunoaffinity can capture specific EV subtypes with high quality at a low cost and within a short period. The activity of captured EVs is preserved to a great extent, and the captured EVs can be directly used for downstream analysis after elution (Tauro et al., 2012; Li et al., 2019). Recently, He et al. developed an immunoaffinity capture method for plant EVs (He et al., 2021). In this method, agarose beads conjugated with TET8 antibodies can efficiently isolate TET8-positive EVs (He et al., 2021). By using this method, several RNA-binding proteins (RBPs), such as argonaute 1 (AGO1), annexin1, and RNA helicases (RH11 and RH37), have been identified in TET8-positive EVs (He et al., 2021).

## Evs in Cross-Kingdom Rna Interference

sRNAs are short noncoding molecules that induce RNA interference (RNAi; Baulcombe, 2004). RNAi is a regulatory mechanism for gene expression that is conserved throughout the domain Eukarya. During microbial infection, host sRNA functions endogenously by regulating gene expression to balance plant immunity and growth (Katiyar-Agarwal and Jin, 2010). Emerging studies have shown that bidirectional sRNA trafficking between hosts and interacting microorganisms/pests silence target genes *in trans* in a mechanism referred to as cross-kingdom RNAi (Cai et al., 2018a; Huang et al., 2019). For example, *Arabidopsis* delivers small interfering RNAs (siRNAs), including phased secondary siRNAs, into interacting *B. cinerea* cells, inducing the silencing of fungal genes that are involved in vesicle trafficking pathways (Cai et al., 2018b). Cross-kingdom RNA trafficking from the host into the pathogen to induce the silencing of corresponding pathogenic genes has also been observed in other plant pathosystems, such as in the cotton—*Verticillium dahliae*, and wheat—*Fusarium graminearum* systems (Zhang et al., 2016; Jiao and Peng, 2018). Pathogens also transfer sRNAs to plant hosts as effectors to promote infection and plant defense (Cai et al., 2021). The trafficking of *B. cinerea* sRNA into *Arabidopsis* cells suppresses host immune genes by using the host RNAi machinery component AGO1 (Weiberg et al., 2013). Moreover, the translocated sRNAs of *Hyaloperonospora arabidopsidis* associate with the host *Arabidopsis* AGO1/RISC to regulate plant host defense genes (Dunker et al., 2020). *Puccinia striiformis* f. sp. *Tritici* (*Pst*), one of the most destructive pathogens of wheat (*Triticum aestivum* L.), produces the microRNA-like RNA 1 to silence the wheat *pathogenesis-related 2* gene, which impairs wheat defenses during wheat–Pst interactions (Wang et al., 2017).

Further studies have revealed that cross-boundary sRNA trafficking depends on EVs (Buck et al., 2014; Cai et al., 2018b, 2021; Hou et al., 2019). sRNA trafficking between cells *via* EVs has been studied in mammalian cells (Valadi et al., 2007; Colombo et al., 2014). In animal systems, the parasite nematode *Heligmosomoides polygyrus* delivers sRNAs *via* EVs into mouse gut epithelial cells to modulate host innate immunity (Buck et al., 2014). However, whether hosts use EVs to send sRNAs into interacting pathogen/parasite cells has long remained unclear. A recent study has revealed that more than 70% of the *Arabidopsis* sRNAs transported into *B. cinerea* cells are present in plant EVs, indicating that in plants, EV-mediated transport is one of the major pathways for the cross-kingdom trafficking of sRNA (Cai et al., 2018b). Furthermore, *Arabidopsis tet8/tet9* double mutants transfer less sRNAs into fungal cells and enhance susceptibility to *B. cinerea* challenge, suggesting that plant EVs contribute to plant immunity by cross-kingdom RNAi (Cai et al., 2018b). This finding was supported by the recent study where in *Arabidopsis*-derived secondary siRNAs were found in EVs and likely silenced target genes in *P. capsici* during natural infection (Hou et al., 2019). However, how sRNAs are selectively loaded into EVs during vesicle biogenesis is poorly understood. A recent study illustrated that plant TET8 positive-EVs contain a variety of RBPs, including AGO1, RHs, and ANNs (He et al., 2021). These RBPs bind to sRNAs to load sRNAs into plant EVs (He et al., 2021). Interestingly, AGO1, RH11, and RH37 selectively load sRNAs into EVs, whereas ANN1/2 bind to RNA nonspecifically, indicating that they contribute to stabilizing sRNAs in EVs (He et al., 2021).

Fungal and bacterial RNA cargoes in EVs have been shown to play a pivotal role in animal host cells by regulating gene expression and immunity (Munhoz da Rocha et al., 2020). For example, the fatal human fungal pathogen *Cryptococcus gattii* secretes EVs for transferring RNAs to host cells as virulence factors (Bielska et al., 2018). In addition, sRNAs cargos have been detected in EVs derived from several bacterial pathogens, such as *Aggregatibacter actinomycetemcomitans*, *Porphyromonas gingivalis*, *Pseudomonas aeruginosa*, and *Treponema denticola* (Koeppen et al., 2016; Choi et al., 2017). These Gram-negative bacteria-derived EVs are released from the outer membranes and are also named as outer membrane vesicles (OMVs; Nahui Palomino et al., 2021). The bidirectional translocation of sRNAs has been observed in plant–pathogen interactions (Wang et al., 2016). However, studies on RNA in EVs derived from plant fungal and bacterial pathogens have not been reported. The fungal pathogen *B. cinerea* and the oomycete pathogen *H. arabidopsidis* have recently been shown to deliver sRNAs into plant host cells (Weiberg et al., 2013; Dunker et al., 2020). Further studies are needed to determine whether the eukaryotic plant pathogens that deliver RNA species into hosts require EVs.

## Biogenesis and Secretion of Plant Evs

Exosomes are derived from MVB trafficking and finally fuse with the plasma membrane (Pegtel and Gould, 2019). The perimeter membrane of the late endosome buds inward to the endosome lumen, forming ILVs, which lead to the formation of multivesicular endosomes (van Niel et al., 2018; Mathieu et al., 2019). Therefore, they are also considered as MVBs. Two major mechanisms of ILV formation exist in animals: Endosomal Sorting Complex Required for Transport (ESCRT)-mediated pathway and ceramide-mediated pathway (van Niel et al., 2018; Mathieu et al., 2019). Mechanisms that drive the mobilization of secretory MVBs and fusion with the plasma membrane require the participation of Rab family proteins (Rab11, Rab35, and Rab27), and SNARE family proteins (vesicle-associated membrane protein 7 and YKT6 V-SNARE homolog; Hsu et al., 2010; Ostrowski et al., 2010; Kowal et al., 2014; Tian et al., 2020; Ferro et al., 2021). In fact, in animal cells, MVBs also fuse with autophagosomes and further form amphisomes following the release of EVs containing autophagy components (Klionsky et al., 2014; Jeppesen et al., 2019). However, the mechanism of the formation of plant EVs remains unclear. The fusion of MVBs with the plasma membrane during plant biotic stress responses or plant growth has been demonstrated (An et al., 2006a,b; Wang et al., 2011; Cai et al., 2018b). During the infection of barley (*Hordeum vulgare*) by powdery mildew fungus (*B. graminis* f. sp. *hordei*), multivesicular compartments fuse with the plasma membrane and then release paramural vesicles that are similar to exosomes and may participate in papilla deposition (An et al., 2006a,b). In lily and tobacco pollen tubes, vacuolar sorting receptors (VSRs) mediate the vacuolar transport of soluble cargoes *via* MVBs and localize to the plasma membrane, indicating that VSR proteins have an additional role in mediating protein transport to the plasma membrane (Wang et al., 2011). A recent study has demonstrated that sphingolipids in plant EVs mainly comprise pure glycol inositol phosphate ceramides (GIPCs; Liu et al., 2020a). Furthermore, the *Arabidopsis tet8* mutant has a low amount of cellular GIPCs and secretes few EVs, suggesting that GIPCs may play a part in the biogenesis of EVs (Liu et al., 2020a).

## Novel Ev- and Rnai-Based Tools For Crop Protection

Host-induced gene silencing (HIGS) is an effective strategy for developing resistant varieties by expressing double-stranded RNAs (dsRNAs) targeting pathogen genes in plants to induce the silencing of essential pathogen genes (Nowara et al., 2010). For example, expressing a dsRNA targeting the *Fusarium verticillioides* gene *gus* enhances tobacco resistance to fungal pathogens (Tinoco et al., 2010). Silencing the *Magnaporthe oryzae* transcription factor *MoAP1* by HIGS in transgenic rice leads to improved blast disease resistance (Guo et al., 2019). HIGS has also been used to inhibit the growth of western corn rootworm (*D. virgifera* LeConte) in corn and rust fungi (*Puccinia triticina*) in wheat (Yin et al., 2011; Bolognesi et al., 2012; Panwar et al., 2013). This strategy also can effectively prevent and control root diseases that are difficult to control *via* traditional chemical control. An example of such a disease is cotton Verticillium wilt caused by *V. dahlia* (Wang et al., 2016). Recently, SmartStax Pro, a genetically modified organism (GMO) crop developed by Bayer on the basis of RNAi technologies against insect pests, has been approved by the US government (Rosa et al., 2018). This GMO can express dsRNA corresponding to rootworm *Snf7* messenger RNA (mRNA; Rosa et al., 2018).

However, HIGS has some disadvantages: (1) Transgenic expression is not always stable and is inhibited or silenced after generations; (2) uncertainty about government approvals and public concerns about GMOs; (3) currently, many plants cannot be genetically modified by transgenic technology. Recent studies have indicated that spraying dsRNAs or sRNAs that target crucial pathogen genes can provide efficient and sustainable protection to plants to solve the above problems (Figure 1). This new and innovative technology is called spray-induced gene silencing (SIGS), which has been applied to control numerous economically important plant pathogens (Wang et al., 2016; Cai et al., 2018a). For example, spraying the long noncoding dsRNA *CYP3*, which targets three fungal cytochrome P450 lanosterol C-14α-demethylases, inhibits the growth of *F. graminearum* on barley (Koch et al., 2016). Spraying dsRNA and sRNAs that target *B. cinerea Dicer-like 1* and *2* on the surfaces of fruits, vegetables, and flowers can effectively inhibit gray mold disease caused by *B. cinerea* (Wang et al., 2016). *H. arabidopsidis* is an obligate biotrophic oocyte pathogen that induces downy mildew in *Arabidopsis*. The application of exogenous sRNA or dsRNAs synthesized *in vitro* and targeting the conserved *cellulose synthase A3* gene of *H. arabidopsidis* impairs spore germination and hence prevents the infection of *Arabidopsis* (Bilir et al., 2019).

The success of SIGS for plant disease management is largely determined by RNA uptake efficiency, which varies among different pathogens. Some eukaryotic microbes, including *B. cinerea*, *Rhizoctonia solani*, *Aspergillus niger*, and *V. dahliae*, could uptake environmental RNA efficiently, whereas RNA uptake is modest in *Trichoderma virens*, undetectable in *Colletotrichum gloeosporioides*, and limited in *P. infestans* (Qiao et al., 2021). The efficiency of SIGS application also depends on RNA stability in the environment. Many studies have shown that EVs can protect sRNAs from degradation in the environment and have high uptake efficiency by host cells (Colombo et al., 2014; Liu et al., 2020b). Liposomes, also called artificial vesicles, are spherical vesicles that are encased by a lipid bilayer with nontoxicity, low immunogenicity, and high biocompatibility (Tseng et al., 2009). Liposomes and lipid-based nanoparticles are the most advanced and potent delivery systems for RNA drugs, which can effectively deliver siRNAs to their targets, reducing total siRNA doses and thus attenuating their potential toxicity (Ickenstein and Garidel, 2019). For therapeutic applications in mammalian systems, EVs and artificial vesicles not only carry RNAs but also carry other beneficial molecules, such as celastrol and curcumin for anticancer therapy and gold nanoparticles for improved imaging (Aqil et al., 2016; Meng et al., 2020). Current studies in the field of plant–pathogen interaction have shown that plants use EVs to transport sRNAs into their fungal pathogens (Cai et al., 2018b). Given this new knowledge, incorporating RNAs into artificial vesicles/liposomes or nanoparticles will likely facilitate RNA delivery *via* SIGS approaches to protect RNAs from degradation or water rinsing (Figure 1). Indeed, dsRNA loaded on nanoparticles, such as nontoxic layered double hydroxide clay nanosheets (BioClay), can continuously protect plants against virus even after 30days of spraying (Mitter et al., 2017). Thus, the use of EVs and nanoparticles as carriers of RNAi for crop protection is a promising field in the future.

## Concluding Remarks and Future Perspectives

In the past few decades, EVs have been considered as effective carriers for intercellular communication in prokaryotes and eukaryotes due to their capability to transfer proteins, lipids, nucleic acids, and other biologically active substances, thus affecting a variety of the physiological and pathological functions of their receptor cells (Colombo et al., 2014). In recent years, many studies have revealed that EVs are crucial tools for communication between plants and pathogens and execute considerable functions in host immunity and pathogen virulence (Cai et al., 2021). Plant cells secrete EVs containing sRNAs into fungal cells to induce the silencing of fungal genes that are critical for pathogenicity (Cai et al., 2018b). Recent studies have shown that EVs from the cotton pathogen *Fusarium oxysporum* f. sp. *vasinfectum* induce a phytotoxic response in plants (Bleackley et al., 2019). In addition, OMVs derived from bacteria play a vital part in biofilm formation, virulence, and plant immune regulation during plant–bacterium interactions (Katsir and Bahar, 2017). However, reports on RNAs in EVs derived from plant pathogens or interacting microbes still not exist. Further studies need to be performed to determine whether plant pathogen EVs contain RNAs that are functional within host plant cells.

RNA interference is a conserved biological defense mechanism and is thus an effective method for controlling a variety of pests and pathogens (Majumdar et al., 2017; Zhang et al., 2017). sRNAs can move between interacting organisms, inducing gene silencing in each other, in a process called cross-kingdom RNAi (Cai et al., 2018a). HIGS by the transgenic expression of pathogen dsRNA is thus expected to be an important disease control method (Rosa et al., 2018). Given the disadvantages of transgenic approaches, sRNAs can be directly sprayed on host plants or postharvest products to silence target pathogenic genes in an approach known as SIGS (Cai et al., 2018a). Such an approach can provide effective and sustainable protection to plants. However, the success of SIGS for plant disease management largely depends on the efficiency of dsRNA uptake, which varies among different pathogens (Qiao et al., 2021). The stability of RNA in the environment also affects the efficiency of SIGS (Mitter et al., 2017). Strikingly, EVs can protect sRNAs from environmental degradation and can be efficiently absorbed by host cells (Colombo et al., 2014; Liu et al., 2020b). Artificial vesicles and lipid-based nanoparticles can effectively send siRNAs to their targets at low overall doses and with low potential toxicity (Ickenstein and Garidel, 2019). Therefore, the combined use of artificial vesicles/nanoparticles and SIGS is a desirable method for crop protection.

The current research on the role of EVs in plant immune response remains in its infancy. Many momentous molecular mechanisms of EVs, such as biogenesis (MVB formation and secretion), and receptor cell absorption, remain to be further investigated. In addition to siRNAs, whether long noncoding RNAs and mRNAs exist in plant EVs is unclear. Additional plant EV cargos and their functions require urgent confirmation. In addition, due to the heterogeneity of EVs and their complex functions, further isolating different EVs and determining the effect of each type of EV are very meaningful. He et al. developed an immunoaffinity capture-based technique that can accurately isolate the subtypes of specific EVs (He et al., 2021). Different types of EVs can be obtained *via* direct immunoaffinity capture by using specific antibodies to determine the components and subsequent functions of each kind of vesicle. Studying and identifying additional EV markers in plants is the most current urgent task. Undoubtedly, EVs are a treasured land to seek.

## Author Contributions

GL reviewed the published data and prepared the initial draft. GK, SW, and YH collaborated in manuscript preparation. QC designed the structure of the review, supervised the manuscript preparation and revised and finalized the manuscript. All authors read and approved the final manuscript.

## Funding

Work in the Q.C. laboratory was supported by grants from the National Natural Science Foundation of China (32070288).

## Conflict of Interest

The authors declare that the research was conducted in the absence of any commercial or financial relationships that could be construed as a potential conflict of interest.

## Publisher’s Note

All claims expressed in this article are solely those of the authors and do not necessarily represent those of their affiliated organizations, or those of the publisher, the editors and the reviewers. Any product that may be evaluated in this article, or claim that may be made by its manufacturer, is not guaranteed or endorsed by the publisher.
